# Spatial dynamics of the COVID-19 pandemic in Brazil

**DOI:** 10.1017/S0950268821000479

**Published:** 2021-02-25

**Authors:** R. R. Castro, R. S. C. Santos, G. J. B. Sousa, Y. T. Pinheiro, R. R. I. M. Martins, M. L. D. Pereira, R. A. R. Silva

**Affiliations:** 1 Postgraduate program in Clinical Nursing Care and Health, Universidade Estadual do Ceará, Fortaleza, Ceará, Brasil; 2 Postgraduate program in Nursing, Faculdade Metropolitana de Ciências e Tecnologia, Parnamirim, Rio Grande do Norte, Brasil; 3 Faculdade Maurício de Nassau, João Pessoa, Paraíba, Brasil; 4 Faculdade Santo Antônio de Caçapava, São Paulo, Brasil; 5 Postgraduate program in Clinical Care in Nursing and Health, Universidade Estadual do Ceará, Fortaleza, Ceará, Brasil; 6 Postgraduate program in Nursing, Universidade Federal do Rio Grande do Norte, Natal, Rio Grande do Norte, Brasil; 7 Postgraduate program in Collective Health, Universidade Federal do Rio Grande do Norte, Natal, Rio Grande do Norte, Brasil

**Keywords:** Brazil, COVID-19, pandemic, spatial analysis

## Abstract

The objective of this study was to analyse the dynamics of spatial dispersion of the coronavirus disease 2019 (COVID-19) in Brazil by correlating them to socioeconomic indicators. This is an ecological study of COVID-19 cases and deaths between 26 February and 31 July 2020. All Brazilian counties were used as units of analysis. The incidence, mortality, Bayesian incidence and mortality rates, global and local Moran indices were calculated. A geographic weighted regression analysis was conducted to assess the relationship between incidence and mortality due to COVID-19 and socioeconomic indicators (independent variables). There were confirmed 2 662 485 cases of COVID-19 reported in Brazil from February to July 2020 with higher rates of incidence in the north and northeast. The Moran global index of incidence rate (0.50, *P* = 0.01) and mortality (0.45 with *P* = 0.01) indicate a positive spatial autocorrelation with high standards in the north, northeast and in the largest urban centres between cities in the southeast region. In the same period, there were 92 475 deaths from COVID-19, with higher mortality rates in the northern states of Brazil, mainly Amazonas, Pará and Amapá. The results show that there is a geospatial correlation of COVID-19 in large urban centres and regions with the lowest human development index in the country. In the geographic weighted regression, it was possible to identify that the percentage of people living in residences with density higher than 2 per dormitory, the municipality human development index (MHDI) and the social vulnerability index were the indicators that most contributed to explaining incidence, social development index and the municipality human development index contributed the most to the mortality model. We hope that the findings will contribute to reorienting public health responses to combat COVID-19 in Brazil, the new epicentre of the disease in South America, as well as in other countries that have similar epidemiological and health characteristics to those in Brazil.

## Introduction

The coronavirus disease 2019 (COVID-19), initially identified in China, in December 2019 [1], is a highly transmissible infectious disease that has spread rapidly throughout the world. In South America, the first case of the disease was registered in Brazil, on 26 February 2020 [2]. Due to the outbreak on all continents, the World Health Organization (WHO) assessed that COVID-19 could be characterised as a pandemic on 11 March 2020. In Brazil, cases continued to increase and just over four months after the first case, the country had accumulated more than 2 million confirmed cases and 80 thousand deaths [3].

On the basis of this context, the pandemic situation presents itself as a challenge for health authorities and requires measures to be implemented resulting in the control of the spread of COVID-19 [4]. Therefore, understanding the aspects related to transmissibility is essential for disease control and harm reduction [4].

Recent studies [5–10] analysed the dispersion of COVID-19 in some Brazilian municipalities and observed specific flows in each analysed location. Furthermore, it is possible to notice that these flows can be influenced by factors related to socioeconomic, cultural and health inequalities [8]. Then, it is understood that COVID-19 presents a syndemic nature. The term syndemic refers to biologic and social interactions that are important to establish prognosis, treatment, public policy and social protection. In the case of COVID-19, social and biological determinants of health may influence the incidence of the disease [11].

Taking into account the different standards of inequality presented by various regions of Brazil [12], an analysis of the patterns of dispersion throughout the national territory − not limited to specific regions − is fundamental in the identification of elements that can help understand the current health situation and its impacts in varied scenarios [12]. This approach seeks to facilitate the decision-making process regarding the most effective strategies in controlling the disease.

Spatial analysis methods are increasingly efficient for the identification of areas of greatest risk and, consequently, provide support for the implementation of control measures. These measures, once implemented, can serve as an example for other countries that have the same profile and the same transmission dynamics as Brazil.

Thus, this study is relevant as it permits the observation of possible spatial conglomerates of COVID-19 throughout Brazil, as well as the identification of areas of greatest risk. Furthermore, it contributes to the pandemic by providing information that supports preventive action, control and treatment, reduction of mortality including defining priorities. Therefore, this study aims to analyse the patterns of spatial dispersion of incidence and mortality from COVID-19 in Brazil by correlating them to socioeconomic indicators.

## Methods

**Fig. 1.**
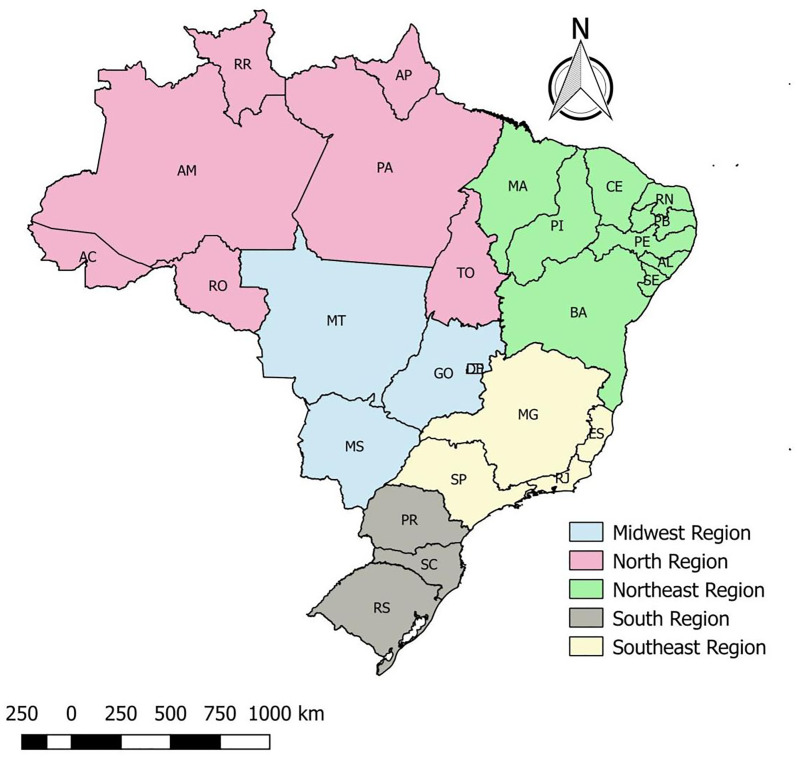
Distribution of states in Brazilian regions, Brazil, 2020.

This is an ecological study, which uses municipalities throughout the Brazilian territory as units of analysis. Brazil is a country in South America, it possesses 5570 municipalities distributed across 26 states and one federal district (Fig. 1), it has an estimated population of 211 757 141 inhabitants, an area of 8 510 295 914 km^2^ and a demographic density of 22.43 inhabitants/km^2^ [13].

### Data collection and analysis

For this study, the data used were provided by the Brazilian Health Department through the electronic website where all data and indicators referring to COVID-19 in the country are available as public domain [3]. The federal database comprised all cases tested and confirmed for the disease from 26 February to 31 July 2020.

Initially, the incidence of the disease in the country was calculated, using the number of cases accumulated in each municipality as the numerator and the population of this municipality estimated in 2019 by the Brazilian Institute of Geography and Statistics [13]. Moreover, the disease mortality was calculated with the number of deaths in the numerator and the population of each municipality in the denominator. The constant for both indicators was 100 000 inhabitants.

### Descriptive analysis

For the spatial analysis, a thematic map of the incidence and mortality of COVID-19 in each municipality was created, then, these rates were smoothed through the local empirical Bayesian method to reduce the instabilities caused by the differences between each city. This method considers not only the value of the municipality but weighs it in the relation of borders between municipalities through a spatial proximity matrix, taking into account the contiguity criterion in which the value of 1 is attributed to the municipalities that share borders in and 0 to municipalities that do not share borders.

### The global and the local Moran index analysis

After the descriptive analysis, the presence of spatial dependence was verified through the global Moran index on the gross indicator using the incidence and mortality rates of COVID-19 of Brazilian municipalities. The method identifies spatial autocorrelation and varies between −1 and +1, where values close to zero indicate the absence of spatial dependence, considering significant *P* < 0.05. Also, the local autocorrelation (local index of spatial association – LISA) was evaluated by the local Moran index, which verifies the value of the municipality with that of its neighbours with identification of spatial patterns [14].

The local Moran index identifies four quadrants: high−high (municipalities with high rates and surrounded by those with high rates), low−low (municipalities with low rates surrounded by those with low rates), high−low (municipalities with high rates surrounded by those with low rates) and low−high (municipalities with low rates surrounded by those with high rates), taking into account values with *P* < 0.05 as significant. The high−high and low−low categories represent areas of conformity and the high−low and low−high categories indicate areas of epidemiological transition [14].

### Regression analysis

After the descriptive spatial analysis, we sought to identify which characteristics were related to this pattern. Thus, a multivariate linear regression analysis was performed by using the incidence and mortality due to COVID-19 as outcomes. The following indicators were considered predictors: illiteracy rate in people over 18 years old, Gini index, average income per capita, percentage of the population living in households with a density greater than two people per bedroom, proportion of the population in the household with bathroom and running water, social vulnerability index (SVI), municipality human development index (MHDI), demographic density and coverage of primary health care. These indicators were taken from the Brazil atlas that consolidates socioeconomic, demographic and vulnerability information for each municipality through data from the last Brazilian census [15]. It is noteworthy that of the 5570 Brazilian municipalities, five did not contain the data described above and were removed from the analyses.

The variables that presented *P* < 0.05 remained in the final model. After this stage, the analysis of the regression residuals was performed to verify the presence of spatial dependence. Once verified, the geographically weighted regression (GWR) model was used as it considers a spatial component as an important factor in the model [16].

In this sense, it is important to differentiate OLS and GWR models. The OLS models are considered global models, which investigate the relationship of a set of independent variables according to an outcome. This type of model does not consider the geographic distribution of the analysed event, and may not be the most appropriate model to the dataset of COVID-19. This model, however, can be used as a base model to compare to other ones [17].

Since OLS models do not take the neighbour cases into account, a GWR model overcomes this obstacle by applying a contiguity matrix. Therefore, it is capable of analysing events that vary over the studied area; because of that, GWR is called a local model. It is important to highlight that GWR uses the principle of heterogeneity and non-stationarity in space, and it generates a regression coefficient for each one of the geographic units that are in analysis. In the case of this study, the coefficients are for each municipality of Brazil and, then, it can be analysed where the indicator is a risk or a protective factor [17, 18].

Nevertheless, some studies have already shown the importance of working with local geographical models in the COVID-19 scenario. Literature shows that such a model was used to determine the influence of sociodemographic indicators on incidence and mortality of COVID-19 in European countries and the United States [17, 18]. At a Brazilian level, mortality by the disease was modelled by applying GWR in São Paulo [19]. Therefore, it is possible to emphasise that such a model can be used in cases of people infected by severe acute respiratory syndrome-coronavirus disease-2.

To assess which of the models was the most appropriate, the Akaike criterion (AIC) and the determination coefficient *R*^2^ were used. Thus, the one with the lowest AIC value and the highest *R*^2^ was considered as the best model [20].

Moreover, models were also evaluated regarding multicollinearity by the variance inflation factors (VIF). Therefore, variables that, after regression, presented VIF >10 are considered with multicollinearity and should be removed.

The calculations of the gross and smoothed rate as well as the Moran spread index and its significance were developed in the TerraView 4.2.2 software. All maps were produced using the QGIS 2.4.17 software. The analyses were performed in the software Stata13, GeoDa 1.14.0 e GWR 4.0.

This study was not submitted to the Research Ethics Committee due to the utilisation of Brazilian public domain databases of COVID-19, hence available on the websites of the Federal Government. It is important to note that it is not possible to identify the patient, as no such information has been made available.

## Results

### Spatial autocorrelation analysis of the incidence and mortality rate

**Fig. 2.**
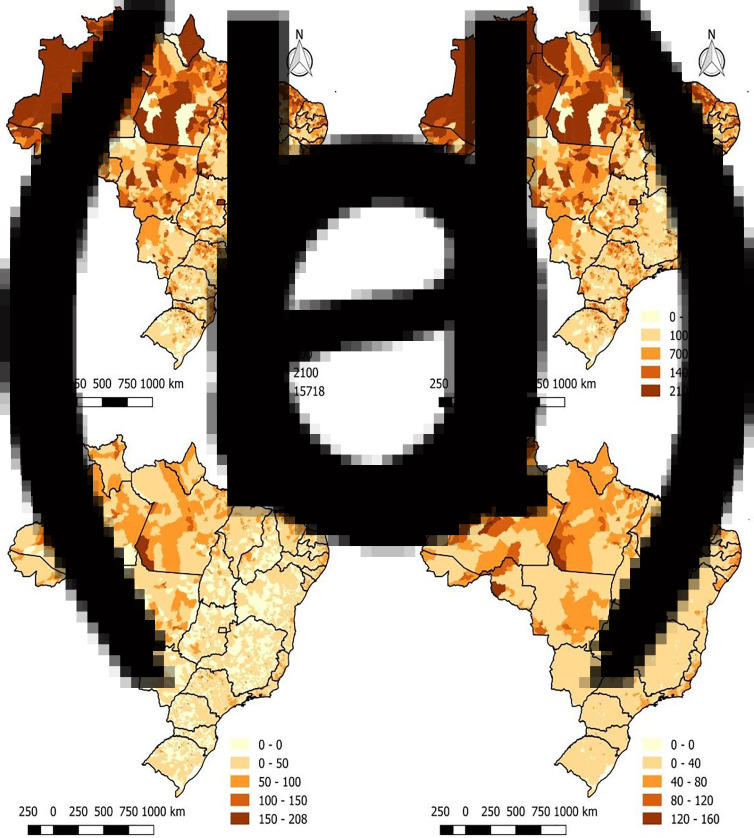
Spatial distribution of the gross and Bayesian, Moran global and local spatial autocorrelation indicators of the Covid-19 cases in Brazil, 26 February to 31 July 2020. (a) Gross rate of new cases (b) Bayesaian rate of new cases (c)Crude mortality rate (d) Bayesian mortality rate.

From February to July 2020, 2,662,485 confirmed cases of COVID-19 were reported in Brazil. Figure 2a demonstrates that most Brazilian municipalities had at least one case of COVID-19 since only 123 did not register any cases. The highest incidence rates in the country reach maximum values in states in the north and northeast region. Through the approach of the local empirical Bayesian method (Fig. 2b), it is possible to notice that the rates were weighted, and the areas showed a better distribution of the incidence. However, the municipalities have similar gross and Bayesian rates, but with uniformity in the central region of the country.

In the spatial autocorrelation analysis of the incidence rate, the global Moran index was 0.50, with *P* = 0.01, indicating a positive spatial autocorrelation. Through the local Moran index, spatial clusters can be identified (Fig. 2), where the main high−high patterns are found in the north, northeast and the largest cities in the southeast. The main low−low clusters are concentrated in central Brazil. In the LISA map, it is possible to see the significance of each of these clusters (Fig. 2d).

**Fig. 3.**
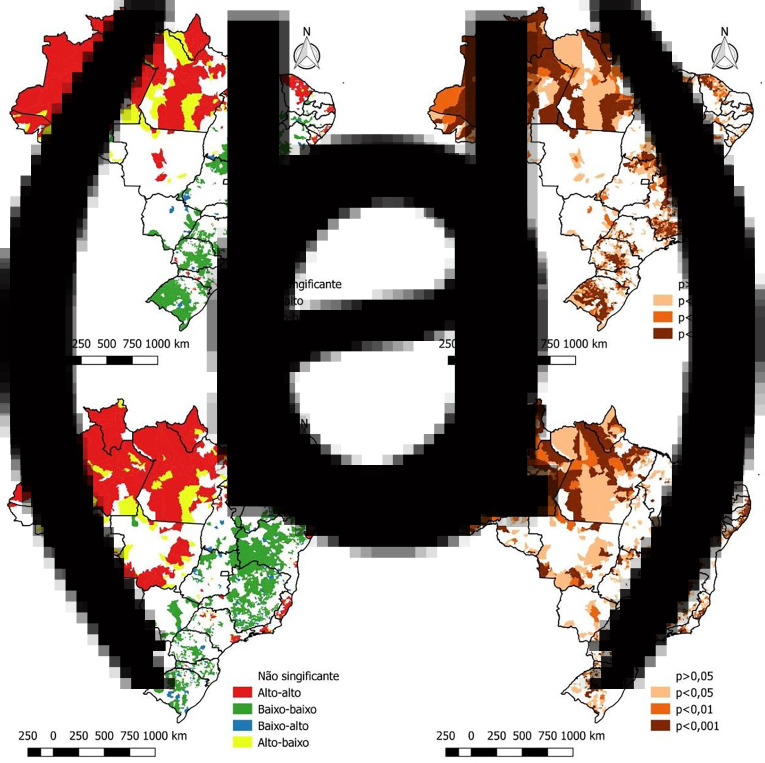
Spatial distribution of crude and Bayesian spatial autocorrelation, Moran global and location of COVID-19 mortality in Brazil, from 26 February to 31 July 2020. (a) Moran map of new cases (b) Lisa significance map of new cases (c) Moran map of mortality (d) Lisa significance map of mortality.

Regarding the mortality of the disease, the same period reported 92 475 deaths from COVID-19. Through Figure 3a, it can be seen that the northern states of Brazil, mainly Amazonas, Pará and Amapá, presented high mortality, reaching more than 150 deaths per 100 thousand inhabitants. By applying the local empirical Bayesian method, areas with high death rates can be seen more clearly in addition to the north region, such as the states of Ceara and Pernambuco (northeast region), Sao Paulo and Rio de Janeiro (region southeast) also with an elevated mortality rate from the disease (Fig. 3b).

In the spatial autocorrelation analysis of the mortality rate, the global Moran index was equal to 0.45 with *P* = 0.01, indicating a positive spatial autocorrelation. Through the local Moran index, spatial clusters can be identified (Fig. 3c) where the main high−high patterns are found in the states of Amazonas, Pará, Amapá and Roraima (north region), Ceara and Pernambuco (northeast region) as well as Sao Paulo and Rio de Janeiro (southeast region). The main low−low clusters are concentrated in the states of the midwest region (Mato Grosso, Mato Grosso do Sul, Goias and Brasilia) and the south (Parana, Santa Catarina and Rio Grande do Sul) in addition to the states of Bahia, in the northeast and Minas Gerais, in southeastern Brazil. On the LISA map, it is possible to see the significance of each of these clusters (Fig. 3d).

### OLS regression analyses for the incidents and mortality

When OLS regression was conducted for the incidence of COVID-19 in Brazil, it was observed that the relationship with the Gini index and with demographic density lost statistical significance (*P* > 0.05). The analysis of OLS residuals showed that they were spatially dependent since *I* = 0.338 (*P* < 0.05), requiring the application of a GWR model. When the GWR model was applied to the statistically significant variables in the OLS model, it was observed that most of the association occurred through the direct relation with the SVI (*β* = 148.01), with the MHDI (*β* = 174.61) and with the proportion of the population with density> 2 (*β* = 3.13).

### Other association analyses

Other relationships observed were the direct relationship with the average income per capita (*β* = 0.25), the proportion of the population at home with a bathroom and running water (*β* = 0.68), coverage of primary health care (*β* = 0.32). There was also an inverse relationship with the illiteracy rate in those over 18 years old (*β* = −0.85). However, all of these relationships must be interpreted with caution as the coefficients are close to zero (Table 1).

**Table 1.** Indicators of incidence of COVID-19

	OLS regression			GWR regression	
Indicators	*β*	Standard error	*P*-value	*β* (mean)	Standard deviation
Illiteracy rate in those over 18 years old	−3.83	0.60	<0.001	−0.85	7.14
Average income per capita	0.21	0.03	<0.001	0.25	0.35
Proportion of the population at home with a bathroom and running water	−1.00	0.28	<0.001	0.68	3.31
Proportion of population with density > 2	9.34	0.40	<0.001	3.13	4.95
SVI	282.68	55.20	<0.001	148.01	430.50
MHDI	187.04	69.75	0.007	174.61	668.74
Coverage of primary health care	0.66	0.14	<0.001	0.32	1.17

The OLS regression for mortality due to COVID-19 in Brazil, on the other hand, showed that the relationship with the proportion of the population at home with bathroom and running water and coverage of primary health care lost statistical significance (*P* > 0.05). The analysis of the residuals showed that they presented spatial dependence because *I* = 0.299 (*P* < 0.05), also showing the need to apply a GWR model. When the GWR model was applied to the statistically significant variables in the OLS model, it was observed that most of the relationship occurred directly with the SVI (*β* = 11.01) and with the MHDI (*β* = 7.47) and inversely with the Gini index (*β* = −8.08).

Other relationships evidenced by the GWR model were directly related to per capita income (*β* = 0.01), the proportion of population with density > 2 (*β* = 0.07) and demographic density (*β* = 0.0004). An inverse relationship was identified with the illiteracy rate in those over 18 years old (*β* = −0.14). As with the incidence, these relationships must be interpreted with caution because the coefficients are close to zero (Table 2).

**Table 2.** Indicators of mortality of COVID-19

	OLS regression			GWR regression	
Indicators	*β*	Standard error	*P*	*β* (mean)	Standard deviation
Illiteracy rate in those over 18 years old	−0.17	0.02	<0.001	−0.14	0.41
Gini index	−9.11	2.37	<0.001	−8.08	14.52
Average income per capita	0.005	0.001	<0.001	0.01	0.01
Proportion of population with density > 2	0.30	0.01	<0.001	0.07	0.19
SVI	16.79	2.16	<0.001	11.01	23.79
MHDI	5.87	2.70	0.03	7.47	25.42
Demographic density	0.002	0.0002	<0.001	0.0004	0.01

Regarding the evaluation of the models, there was a better performance of the GWR model compared to the OLS. This occurs when, for the incidence of COVID-19, it was observed that the OLS model presented AIC = 76 428 and *R*^2^ = 0.201, and the GWR presented AIC = 74 580 and *R*^2^ = 0.450. As for mortality, the AIC = 40 331 and *R*^2^ = 0.151 and the GWR model presented AIC = 38 466 and *R*^2^ = 0.420. In both models, the mean VIF was 3.9, and the variable that most inflates variance was the SVI (VIF = 6.1). It is noteworthy that, once GWR is a local model and each municipality had its regression coefficient, the standard deviation presented a high value. In other words, it may be possible to encounter cities with high *β* and others with low ones.

## Discussion

The study of the spatial distribution of COVID-19 and the relationship with social and health indicators in Brazil made it possible to understand the dynamics of COVID-19 in the first five months of the disease and expand knowledge about the epidemiological chain of the disease in the country. The heterogeneity of the disease corroborates to validate the hypothesis that the incidence and mortality due to COVID-19 are associated with a combination of geographical, economic, social and cultural factors that express the population's way of life.

Our results indicate that the highest incidence rates for COVID-19 occurred in the north and northeast regions and also in large cities in the southeast region (Sao Paulo and Rio de Janeiro) which are configured, respectively, in the regions with the lowest human development indexes (HDI) in the country and the largest urban centres. Regarding mortality rates, the north region stood out, in conjunction with states such as Ceara, Pernambuco, Sao Paulo and Rio de Janeiro. High−high spatial clusters were observed in the north and northeast regions. The midwest and south regions showed a low−low pattern.

COVID-19 has presented itself dynamically on the world stage, reaching developed and underdeveloped countries without distinction [21]. The disease advanced rapidly throughout Brazil, with community transmission declared all through the national territory. As one of the countries with the largest number of cases together with its neighbours in Latin America, it was configured as the new epicentre of the disease in mid-June 2020 [6, 22].

A study developed on the estimation and prediction of COVID-19 cases in Brazil [14], pointed out that the largest number of cases is found in the southeast region, especially in the cities of Sao Paulo and Rio de Janeiro, corroborating the results of this research study. The high number of cases in these cities is because they conform as being the main economic and tourist regions of the country. Sao Paulo is the main financial, corporate and commercial centre in South America, moreover, Rio de Janeiro is the largest international tourist destination in the country, contributing to a greater circulation of people from abroad, consequently, increasing the emergence of cases of the disease [6, 21].

Despite registrations in almost all municipalities, the spatial distribution of COVID-19 cases was heterogeneous in the Brazilian scenario and, although initially related to regions with a wide tourist and commercial network, COVID-19 advanced towards a significant local transmission reaching regions that fled the logic initially established. In the spatial distribution of incidence and mortality, the north and northeast are among the regions with the highest rates, revealing the high risk of infection and mortality. Both regions had municipalities with incidence rates that were two to 10 times higher than the incidence rates of municipalities in the midwest, south and southeast regions.

Epidemiological and spatial analysis studies [23, 24] have revealed that most populous cities have also the highest incidence of COVID-19. In contrast, the results found in Brazil reveal that the north and northeast regions, holding the lowest demographic densities in the country, form the clusters with the highest incidence rates. Nevertheless, these regions have cities with high demographic incidence due to high migratory and tourist flows, thus justifying their high rates. The absence of a relationship between the indication of the disease and the population density observed in the regression analysis can be explained by the fact that the increase in the transmission rate and the incidence depend on the rate of interpersonal contact of a population, regardless of its density [25]. It is worth mentioning that these results need to be analysed with caution.

Another factor on the basis of the capacity of the health system is the national rates of ICU beds per 10 000 inhabitants, where the north and northeast regions have the worst national scores, with 0.9 beds per 10 000 inhabitants in the north and 1.5 per 10 000 inhabitants in the northeastern regions [26]. Besides, the high mortality identified especially in the areas can be justified by conditions related to health care, socioeconomic and demographic conditions, and factors intrinsic to the population (age, presence of comorbidities and life habits) [27–29].

The states of Amazonas, Pará, Amapá and Roraima, in the northern region, and the states of Ceara and Pernambuco, in the northeastern region, stand out for their formations of spatial clusters, thus revealing a risk for the spread of the disease and requiring special attention in control strategies from government authorities [12]. The city of Manaus, capital of Amazonas, stands out, as it represents the largest financial and economic centre of the north region and with important tourist activity. The presence of agglomerates in Manaus and the metropolitan region is linked to a social scenario in which social inequality, competitiveness and fragile relations between municipalities prevail, resulting in an insufficient network of health services and a quantitative deficiency of human resources [21, 30]. The direct relationship of social vulnerability, expressed by the SVI, which verifies population conditions in addition to economic aspects, including housing and education conditions, corroborates to explain the existence of spatial clusters in these regions, since factors that express poverty increase the risk of transmission by infectious diseases [16].

In Brazil, the State of Ceara is the third with the largest number of confirmed cases in the country, with a high increase in COVID-19 cases between February and July 2020 [3, 31]. The state capital, Fortaleza, together with the metropolitan region, presented the formation of spatial agglomeration. It is inferred that the formation of high−high clusters for COVID-19 is related to the economic and social characteristics of the region [21].

In the GWR analysis, the MHDI, a measure composed of indicators of three dimensions of human development, longevity, education and income, showed a direct relationship with incidence and mortality. It is believed that this relationship may be related to the dynamics of the beginning of the pandemic in the country, since, initially, the greatest number of cases was present in places with greater circulation of people from other countries, verified in municipalities with high MHDI values, such as Rio de Janeiro, São Paulo and Fortaleza [24].

Another socioeconomic indicator is the income per capita since municipalities with higher income had a higher incidence and mortality due to COVID-19. It is assumed that the influence of income per capita on the outcomes studied is related to the presence of a better network of health services, expanding the population's access to carrying out diagnostic tests in the municipalities with the highest per capita income. Corroborating this finding, GWR analysis revealed that coverage of primary health care is directly related to the incidence of COVID-19, since expanding access increases the number of diagnosed cases and reduces underreporting, contributing to increasing the coefficients incidence and mortality [16].

The greater socioeconomic vulnerability of the north and northeast regions reverberates in the fight against the disease through prevention and control measures, that contributed to the high incidence and mortality in the region. A study carried out in China revealed that during the first stage of the epidemic, the most economically prosperous cities had more medical resources and obtained lower transmission rates, showing that the greater amount of resources in the health department decreased the transmissibility and mortality of the disease [32].

Another Chinese study presented that there is a relationship between socioeconomic conditions and COVID-19 mortality, showing that the higher the gross domestic product (GDP) per unit of land area and the hospital density, the lower the COVID-19 morbidity rate [32]. Therefore, unstable income, weaknesses in the health system, scarcity of resources and relatively low popular socioeconomic conditions are challenges in containing the pandemic, making these regions more vulnerable to the negative effects of COVID-19 [33, 34].

The high mortality rate in the north region can be related to the marked presence of the indigenous population [35]. Research studies have shown that all people are immunologically susceptible to COVID-19, however, this population is more vulnerable to epidemics due to worse social, economic and health conditions, increasing the spread of diseases [34]. Also, barriers to access health services, such as geographical distance, shortage of human resources in the health and linguistic fields, as well as a lifestyle that allows them to be more exposed to infectious diseases, such as living in collective houses and sharing personal utensils are all contributing factors. Many indigenous people have been victims of COVID-19, but the difficulty in diagnosing and notifying residents in unapproved indigenous areas contributes to underreporting the number of cases and deaths [34–36].

The combination of determining factors such as the occasion when the first cases occurred, densification and population displacement flow; the age distribution; clinical support conditions for critically ill patients; the timing and extent of interventional actions in the communities; available testing capacity and the communication measures adopted by public health agencies to reduce viral spread are intervening elements in the behaviour of the disease in any given region [19].

The use of the Bayesian model, global and local Moran and GWR made it possible to understand the phenomenon of COVID-19 in the Brazilian scenario and to point out the social and economic factors involved in the geographical progression of the disease. By understanding the patterns of the spatial distribution of COVID-19 in Brazil, takes into account that the adoption of differentiated implementation measures and the establishment of strategic intervention schedules in respective social determinants demonstrated positive contributions in the fight against COVID-19.

This study presented some limitations. Firstly, the methods and analysis applied cannot infer causality. Secondly, data may be updated because the last national census was conducted in 2010; a new one was scheduled for this year, but because of COVID-19, it had to be delayed. In this sense, secondary data analysis can be limited by the incompletion of the dataset. Finally, aggregated studies do not consider individual-level variables such as age/sex/race and, therefore, limit conclusions.

## Conclusion

The results presented provided information to identify which geospatial characteristics contribute to a higher incidence and mortality of the disease and to analyse the coping measures adopted based on the expansion of the disease in the country.

This research study indicates that the largest urban centres and spaces with the lowest HDI are the most affected by COVID-19, showing that socioeconomic aspects are directly related to the disease. We hope that our findings can guide public health responses in the fight against COVID-19 in Brazil and other countries that have similarities in the characteristics of spatial dispersion related to sociodemographic aspects.

## Data Availability

The data that support the findings of this study are available on the Integrated Health Surveillance Platform (Ivis) of the Ministry of Health of Brazil (http://plataforma.saude.gov.br/coronavirus/covid-19/).

## References

[1] Zhu N (2020) A novel coronavirus from patients with pneumonia in China, 2019. The New England Journal of Medicine [Internet] 382, 727–733. Available at http://www.nejm.org/doi/10.1056/NEJMoa2001017. 10.1056/NEJMoa2001017 PMC7092803 31978945

[2] United Nations (2020) Latin America and the Caribbean and the COVID-19 pandemic. Economic and social effects. COVID-19 Special Report [Internet], 1–14. Available at https://repositorio.cepal.org/bitstream/%0Ahandle/11362/45351/1/S2000263_en.pdf%0A.

[3] Brasil (2020) Integrated Health Surveillance Platform (Ivis) of the Ministry of Health of Brazil. Brasilia: Ministry of Health [Internet], 1. Available at http://plataforma.saude.gov.br/.

[4] de Jesus JG (2020) Importation and early local transmission of COVID-19 in Brazil, 2020. Revista do Instituto de Medicina Tropical de São Paulo [Internet] 62, e30. doi: http://www.scielo.br/scielo.php?script=sci_arttext&pid=S0036-46652020000100218&tlng=en. 10.1590/S1678-9946202062030 PMC7232955 32401959

[5] Andrade LA (2020) Surveillance of the first cases of COVID-19 in Sergipe using a prospective spatiotemporal analysis: the spatial dispersion and its public health implications. Revista da Sociedade Brasileira de Medicina Tropical [Internet] 53, e20200287. doi: http://www.scielo.br/scielo.php?script=sci_arttext&pid=S0037-86822020000100641&tlng=en. 10.1590/0037-8682-0287-2020 PMC7269533 32491098

[6] Cavalcante JR and de Abreu A de JL (2020) COVID-19 no município do Rio de Janeiro: análise espacial da ocorrência dos primeiros casos e óbitos confirmados. Epidemiologia e Serviços de Saúde [Internet] 29(3), e2020204. doi: https://www.scielo.br/scielo.php?script=sci_arttext&pid=S2237-96222020000300302&lng=pt&nrm=iso&tlng=pt. 10.5123/S1679-49742020000300007 32520107

[7] Fortaleza CMCB (2020) Taking the inner route: spatial and demographic factors affecting vulnerability to COVID-19 among 604 cities from inner São Paulo State, Brazil. Epidemiology & Infection [Internet] 148, e118. Available at https://www.cambridge.org/core/product/identifier/S095026882000134X/type/journal_article. 10.1017/S095026882000134X PMC7324662 32594926

[8] de Souza CDF (2020) Spatiotemporal evolution of coronavirus disease 2019 mortality in Brazil in 2020. Revista da Sociedade Brasileira de Medicina Tropical [Internet] 53, e20200282. doi: http://www.scielo.br/scielo.php?script=sci_arttext&pid=S0037-86822020000100912&tlng=en. 10.1590/0037-8682-0282-2020 PMC7269530 32491106

[9] Pedrosa NL and de Albuquerque NLS (2020) Análise Espacial dos Casos de COVID-19 e leitos de terapia intensiva no estado do Ceará, Brasil. Ciência e Saúde Coletiva [Internet] 25, 2461–2468. Available at http://www.scielo.br/scielo.php?script=sci_arttext&pid=S1413-81232020006702461&tlng=pt. 10.1590/1413-81232020256.1.10952020 32520290

[10] Dornels Freire de Souza C (2020) Spatiotemporal evolution of case fatality rates of COVID-19 in Brazil, 2020. Jornal Brasileiro de Pneumologia [Internet] 46, e20200208–e20200208. Available at http://www.jornaldepneumologia.com.br/detalhe_artigo.asp?id=3362. 10.36416/1806-3756/e20200208 PMC7567637 32578681

[11] Horton R (2020) Offline: COVID-19 – a reckoning. The Lancet [Internet] 395, 935. Available at https://linkinghub.elsevier.com/retrieve/pii/S0140673620306693. 10.1016/S0140-6736(20)30669-3 PMC7156226 32199478

[12] Lana RM . (2020) Emergência do novo coronavírus (SARS-CoV-2) e o papel de uma vigilância nacional em saúde oportuna e efetiva. Cadernos de Saúde Pública [Internet] 36(3), e00019620. doi: http://www.scielo.br/scielo.php?script=sci_arttext&pid=S0102-311X2020000300301&tlng=pt. 10.1590/0102-311x00019620 32187288

[13] Brasil (2020) Cidades IBGE [Internet]. Instituto Brasileiro de Geografia e Estatística. [cited 2020 Dec 14]. Available at https://cidades.ibge.gov.br.

[14] Monteiro LD (2015) Spatial patterns of leprosy in a hyperendemic state in Northern Brazil, 2001-2012. Revista de Saúde Pública [Internet] 49(84), 1–8. doi: http://www.scielo.br/scielo.php?script=sci_arttext&pid=S0034-89102015000100265&lng=en&tlng=en. 10.1590/S0034-8910.2015049005866 PMC4650934 26603352

[15] Brasil (2010) Censo Demográfico 2010. Características da população e dos domicílios: resultados do universo. Instituto Brasileiro de Geografia e Estatística.

[16] de Souza CDF (2020) Modelagem espacial da hanseníase no estado da Bahia, Brasil (2001–2015) e determinantes sociais da saúde. Ciência e Saúde Coletiva [Internet] 25, 2915–2926. Available at http://www.scielo.br/scielo.php?script=sci_arttext&pid=S1413-81232020000802915&tlng=pt. 10.1590/1413-81232020258.21522018 32785529

[17] Mollalo A, Vahedi B and Rivera KM (2020) GIS-based spatial modeling of COVID-19 incidence rate in the continental United States. Science of the Total Environment [Internet] 728, 138884. Available at https://linkinghub.elsevier.com/retrieve/pii/S0048969720324013. 10.1016/j.scitotenv.2020.138884 PMC7175907 32335404

[18] Sannigrahi S (2020) Examining the association between socio-demographic composition and COVID-19 fatalities in the European region using spatial regression approach. Sustainable Cities and Society [Internet] 62, 102418. Available at https://linkinghub.elsevier.com/retrieve/pii/S2210670720306399. 10.1016/j.scs.2020.102418 PMC7395296 32834939

[19] Urban RC and Nakada LYK (2020) GIS-based spatial modelling of COVID-19 death incidence in São Paulo, Brazil. Environment and Urbanization [Internet], 095624782096396. Available at http://journals.sagepub.com/doi/10.1177/0956247820963962. 10.1177/0956247820963962 PMC7557234 38603029

[20] Luc Anselin (2005) *Exploring Spatial Data with GeoDaTM: A Workbook*. 1st ed. U-C U of I, editor. California: Center for Spatially Integrated Social Science, 2–244 p.

[21] Sousa GJB (2020) Estimation and prediction of COVID-19 cases in Brazilian metropolises. Revista Latino-Americana de Enfermagem [Internet] 28, e3345. doi: http://www.scielo.br/scielo.php?script=sci_arttext&pid=S0104-11692020000100365&tlng=en. 10.1590/1518-8345.4501.3345 PMC7319758 32609282

[22] CDC COVID-19 Response Team (2020) Geographic differences in COVID-19 cases, deaths, and incidence – United States. The Morbidity and Mortality Weekly Report [Internet] 69:465–471. Available at 10.15585/mmwr.mm6915e4externalicon. PMC7755058 32298250

[23] Tang Y and Wang S (2020) Mathematic modeling of COVID-19 in the United States. Emerging Microbes & Infections [Internet] 9, 827–829. Available at https://www.tandfonline.com/doi/full/10.1080/22221751.2020.1760146. 10.1080/22221751.2020.1760146 PMC7241447 32338150

[24] Mukherjee K (2020) COVID-19 and lockdown: insights from Mumbai. Indian Journal of Public Health 64, 168–171. Available at http://pubmed.ncbi.nlm.nih.gov/32496249/. 32496249 10.4103/ijph.IJPH_508_20

[25] Hu H, Nigmatulina K and Eckhoff P (2013) The scaling of contact rates with population density for the infectious disease models. Mathematical Biosciences [Internet] 244, 125–134. Available at https://linkinghub.elsevier.com/retrieve/pii/S0025556413001235. 10.1016/j.mbs.2013.04.013 23665296

[26] Associação de Medicina Intensiva Brasileira (2020) AMIB apresenta dados atualizados sobre leitos de UTI no Brasil. Associação de Medicina Intensiva Brasileira [Internet]. Avaliable at https://www.amib.org.br/fileadmin/user_upload/amib/2020/abril/28/dados_uti_amib.pdf

[27] de Cobre AF . (2020) Risk factors associated with delay in diagnosis and mortality in patients with COVID-19 in the city of Rio de Janeiro, Brazil. Ciência & Saúde Coletiva [Internet] 25, 4131–4140. Available at http://www.scielo.br/scielo.php?script=sci_arttext&pid=S1413-81232020006804131&tlng=en. 10.1590/1413-812320202510.2.26882020 33027349

[28] Garnelo L, Sousa ABL and Silva C de O da (2017) Regionalização em Saúde no Amazonas: avanços e desafios. Ciência & Saúde Coletiva [Internet] 22, 1225–1234. Available at http://www.scielo.br/scielo.php?script=sci_arttext&pid=S1413-81232017002401225&lng=pt&tlng=pt. 10.1590/1413-81232017224.27082016 28444047

[29] da Silva JB and Muniz AMV (2020) Pandemia do Coronavírus no Brasil: impactos no Território Cearense. Espaço e Economia [Internet] 9(17), 1–20. doi: http://journals.openedition.org/espacoeconomia/10501.

[30] Qiu Y, Chen X and Shi W (2020) Impacts of social and economic factors on the transmission of coronavirus disease 2019 (COVID-19) in China. Journal of Population Economics [Internet] 33, 1127–1172. Available at http://link.springer.com/10.1007/s00148-020-00778-2. 10.1007/s00148-020-00778-2 PMC7210464 32395017

[31] Galea S (2017) Health haves, health have nots, and heterogeneity in population health. The Lancet Public Health [Internet] 2, e388–e389. Available at https://linkinghub.elsevier.com/retrieve/pii/S2468266717301603. 10.1016/S2468-2667(17)30160-3 29253404

[32] Quinn SC and Kumar S (2014) Health inequalities and infectious disease epidemics: a challenge for global health security. Biosecurity and Bioterrorism: Biodefense Strategy, Practice, and Science [Internet] 12, 263–273. Available at http://www.liebertpub.com/doi/10.1089/bsp.2014.0032. 10.1089/bsp.2014.0032 PMC4170985 25254915

[33] Mesa Vieira C (2020) COVID-19: the forgotten priorities of the pandemic. Maturitas [Internet] 136, 38–41. Available at https://linkinghub.elsevier.com/retrieve/pii/S0378512220302346. 10.1016/j.maturitas.2020.04.004 PMC7195319 32386664

[34] Power T (2020) COVID-19 and indigenous peoples: an imperative for action. Journal of Clinical Nursing [Internet] 29, 2737–2741. Available at https://onlinelibrary.wiley.com/doi/abs/10.1111/jocn.15320. 10.1111/jocn.15320 PMC7272911 32412150

[35] Santos RV, Pontes AL and Coimbra Jr CEA (2020) Um “fato social total”: COVID-19 e povos indígenas no Brasil. Cadernos de Saúde Pública [Internet] 36(10), e00268220. doi: http://www.scielo.br/scielo.php?script=sci_arttext&pid=S0102-311X2020001000201&tlng=pt. 10.1590/0102-311X00268220 33027432

[36] Díaz de León-Martínez L (2020) Critical review of social, environmental and health risk factors in the Mexican indigenous population and their capacity to respond to the COVID-19. Science of the Total Environment [Internet] 733, 139357. Available at https://linkinghub.elsevier.com/retrieve/pii/S0048969720328746. 10.1016/j.scitotenv.2020.139357 PMC7215151 32416536

